# Are cost differences between specialist and general hospitals compensated by the prospective payment system?

**DOI:** 10.1007/s10198-017-0935-1

**Published:** 2017-10-23

**Authors:** Francesco Longo, Luigi Siciliani, Andrew Street

**Affiliations:** 1 Department of Economics and Related Studies, University of York, York, UK; 2 Centre for Health Economics, University of York, York, YO10 5DD UK; 3 Department of Health Policy, London School of Economics and Political Science, London, WC2A 2AE UK

**Keywords:** Specialist hospitals, Orthopaedics, Hospital costs, HRG, Tariff, Reference costs, C21, D24, H51, I18

## Abstract

Prospective payment systems fund hospitals based on a fixed-price regime that does not directly distinguish between specialist and general hospitals. We investigate whether current prospective payments in England compensate for differences in costs between specialist orthopaedic hospitals and trauma and orthopaedics departments in general hospitals. We employ reference cost data for a sample of hospitals providing services in the trauma and orthopaedics specialty. Our regression results suggest that specialist orthopaedic hospitals have on average 13% lower profit margins. Under the assumption of break-even for the average trauma and orthopaedics department, two of the three specialist orthopaedic hospitals appear to make a loss on their activity. The same holds true for 33% of departments in our sample. Patient age and severity are the main drivers of such differences.

## Introduction

The prospective payment system (PPS) is commonly used to reimburse hospitals across Organisation for Economic Co-operation and Development (OECD) countries [1]. It is built on a patient classification system that categorises patients into resource homogeneous groups, with each hospital receiving a fixed pre-determined tariff for every patient falling into a given group. This generates incentives for hospitals to contain costs.

In its purest form, a PPS reimburses hospitals only on the basis of the volume and type of patients treated, without taking organisational characteristics into account. Under the German PPS, for example, tariffs do not depend on the hospital’s ownership status or membership to the national insurance programme [2]. In contrast, other PPSs do consider organisational characteristics. In the French PPS, for instance, prices differ for public and private hospitals [3]. In some countries, the PPS provides greater compensation to allow for the costs of specialist care. An example is the PPS of the Lombardy region in Italy, which applies a tariff top-up to all hospitals with ‘high specialisation’ units [4]. In England, hospitals are paid extra if their patients receive specialised care [5].

Some health care systems feature hospitals that specialise in a single specialty, such as cardiology, ophthalmology, or orthopaedics.^1^ Specialisation is an organisational form which is supposed to generate the benefits of the ‘focused factory’, i.e., greater efficiency, quality, and responsiveness [12–14] but not necessarily lower costs. In the US, the Medicare Payment Advisory Commission showed that the costs of specialist hospitals were no lower than the costs of general hospitals. While cardiac hospitals’ costs were not significantly different from general hospitals’, orthopaedic and surgical hospitals had 20% higher inpatient costs. Higher costs were due to more specialised and costly facilities, higher staffing levels, better quality of care, but also excess capacity and low inpatient volumes [15, 16].

Such findings have stimulated empirical research on specialist hospitals’ costs. Barro et al. [17] study the impact of specialist cardiac hospitals on overall expenditure and quality in the US between 1996 and 1999. They find that entry of specialist hospitals reduces expenditure growth without affecting outcomes. Carey et al. [18] investigate the cost efficiency of US specialist hospitals between 1998 and 2004. They find higher levels of inefficiency in orthopaedic and surgical hospitals compared to general hospitals. Kim et al. [9] analyse South Korean specialty orthopaedic hospitals between 2010 and 2012, which are found to apply higher patients’ charges than general hospitals. The authors suggest that such higher charges are due to greater set-up, investment, staffing, and treatment costs.

The present study contributes to this small empirical literature. We investigate the financial viability of specialist orthopaedic hospitals relative to trauma and orthopaedics (T&O) departments in general hospitals in the English National Health Service (NHS). Our primary objective is to test whether costs of specialist orthopaedic hospitals are higher than T&O departments in general hospitals even after accounting for differences in revenues. In other words, we test whether the current PPS covers the costs of specialist orthopaedic hospitals relative to T&O departments in general hospitals.

In England, the majority of hospitals are funded through the national tariff payment system (NTPS).^2^ The NTPS is characterised by two key elements: the healthcare resource groups (HRGs), which classify patients into homogeneous categories based on diagnoses, procedures and some patients characteristics [3]; and the tariffs, which vary by HRG and admission type (elective or non-elective) and reflect the national cost for an HRG averaged across all hospitals [20]. An additional payment for excess bed days is made for patients whose length of stay is beyond a threshold, called the trim point, which also varies by HRG and admission type.^3^ Both the base and excess bed day tariffs are adjusted by the market forces factor (MFF) index to account for exogenous geographical differences in input prices [21]. Tariffs are inflated if the patient receives specialised services under specific HRGs [5].^4^ With such a payment system, specialist hospitals are likely to obtain higher revenues owing to the greater proportion of patients within an HRG who receive a specialised service.

We collect data at HRG level from the NHS reference cost (RC) database for the financial year 2013/14. Such data allow us to analyse the unit cost per patient of every inpatient HRG delivered through the T&O department of each hospital trust (hospital from now on) in the sample.^5^ Our econometric strategy employs four regressions. The first regression provides raw differences in unit costs between specialist orthopaedic hospitals and T&O departments. In a second regression, we compare unit costs after controlling for differential payments (due to different HRGs and other tariff corrections). This is our key model and provides differences in profit margins between the two types of hospital: given that HRG tariffs are fixed, any differences in unit costs after controlling for differences in payment will be reflected in the profit margin. In the third regression, we explain any differences in profit margins (i.e., in costs after controlling for payment) as a function of possible determinants including patient characteristics such as proportion of males, age, socio-economic status, number of diagnoses and procedures, and hospital characteristics such as the salary of doctors, hospital type, scale economies, quality, and geographical location. Our fourth regression examines the heterogeneity in profit margins across specialist hospitals. We estimate these models by weighted least squares (WLS), clustering standard errors within hospitals.

The English NHS includes 141 general hospitals with a T&O department and three specialist orthopaedic hospitals. Although there are few specialist orthopaedic hospitals, they play an important role in the English NHS. They deliver high proportions of specialised services, commonly low-volume but high-cost treatments for patients with complex and rare conditions. Specialist orthopaedic hospitals therefore allow the achievement of a critical mass of clinical expertise to ensure patients receive specialised treatments that produce better health outcomes [22]. For instance, they provide 90% of bone and soft tissue sarcoma surgeries, and 50% of scoliosis treatments. They also perform high proportions of more common, corrective procedures, such as 50% of revision knee replacements and 20% of revision hip replacements [23]. We focus on specialist orthopaedic hospitals because T&O is the specialty with the fourth highest volume of patients, after general medicine, general surgery, and paediatrics. In 2013, 6.7% of all NHS patients were treated in a T&O department.

To the best of our knowledge, this is one of the first attempts to study differences in profit margins between specialist hospitals and departments within general hospitals undertaking similar activities. Previous work focuses on either costs [e.g., [16] ] or revenues [e.g., [9] ]. Our analysis is at the HRG level, rather than patient level, making use of cost data that all English hospitals are required to report annually to the Department of Health (DH). This is a natural choice since payment is also at the HRG level and our focus is on controlling for differences in payment across hospital types. As cost data are available only at HRG-level in most countries, our methodological approach can easily be employed and replicated in future studies, either to compare specialist and general hospitals, or to make other types of comparison, such as between teaching and non-teaching hospitals.

In the next four sections of the paper, respectively, we provide the economic framework, describe the econometric strategy, illustrate the data and some descriptive statistics, and present the results. The final section discusses and concludes.

## Economic framework

Under a PPS, hospitals are funded according to the number and type of patients treated. In the English payment system, the total revenue of hospital $k = 1, \ldots ,K$ for providing HRG $j = 1, \ldots ,J$ amounts to:

1 $$R_{jk} = R_{jk}^{\text{IN}} + R_{jk}^{\text{EB}} = p_{jk}^{\text{IN}} \left( {1 + te_{jk} } \right)y_{jk} + p_{jk}^{\text{EB}} \left( {1 + te_{jk} } \right)q_{jk} ,$$

where $R_{jk}^{\text{IN}}$ is the total *inlier* revenue of hospital *k* for treating patients who have a normal length of stay for their HRG *j*; $R_{jk}^{\text{EB}}$ is the total *excess bed day* revenue of hospital *k* earned for each additional day that patients stay beyond their specific HRG *j’*s trim point; $p_{jk}^{\text{IN}}$ is the HRG *inlier* price received by hospital *k* for treating a patient falling under HRG *j*; $p_{jk}^{\text{EB}}$ is the *per diem* price received by hospital *k* for a single excess bed day produced under HRG *j*; $t$ is the tariff top-up on specialised orthopaedic services, which is a constant proportion across HRGs and hospitals; $e_{jk}$ is the proportion of patients in hospital *k* falling under HRG *j* receiving a specialised orthopaedic treatment; $y_{jk}$ is the number of patients admitted in hospital *k* under HRG *j*;^6^ and $q_{jk}$ is the number of excess bed days produced in hospital *k* under HRG *j*.

The HRG prices $p_{jk}^{\text{IN}}$ and $p_{jk}^{\text{EB}}$ can be written more explicitly as:

2 $$p_{jk}^{\text{IN}} = \left( {\alpha_{j}^{\text{IN}} + b} \right)m_{k} ,$$

3 $$p_{jk}^{\text{EB}} = \left( {\alpha_{j}^{\text{EB}} + b} \right)m_{k} ,$$

where $\alpha_{j}^{\text{IN}}$ is the *inlier* tariff for treating a patient falling under HRG *j*; $\alpha_{j}^{\text{EB}}$ is the *excess bed day* tariff of each excess bed day under HRG *j*. These do not vary by hospital. In contrast, $m_{k}$ is a MFF index capturing exogenous geographical differences in the prices of hospital inputs (staff, land, and buildings) that vary depending on the hospital’s location. Finally, $b$ is a fixed tariff adjustment common across hospitals, such as pay and price inflation or the national efficiency adjustment.

The total cost of hospital *k* for providing HRG *j* is:

4 $$C_{jk} = C_{jk}^{\text{IN}} + C_{jk}^{\text{EB}} = c_{jk}^{\text{IN}} y_{jk} + c_{jk}^{\text{EB}} q_{jk} ,$$

where $C_{jk}^{\text{IN}}$ is the total *inlier* cost of hospital *k* for treating patients under HRG *j* (up to the trim point); $C_{jk}^{\text{EB}}$ is the total *excess bed day* cost of hospital *k* for the excess bed days produced under HRG *j*; $c_{jk}^{\text{IN}}$ is the *inlier* unit cost of hospital *k* for HRG *j*, and $c_{jk}^{\text{EB}}$ is the *per diem* unit cost of hospital *k* for each excess bed day falling under HRG *j*. Since the national tariffs are set equal to the national average cost, we can write them more explicitly as:

5 $$\alpha_{j}^{\text{IN}} = \frac{{\sum\nolimits_{k} {c_{jk}^{\text{IN}} y_{jk} } }}{{\sum\nolimits_{k} {y_{jk} } }}\quad{\text{ and }}\quad\alpha_{j}^{\text{EB}} = \frac{{\sum\nolimits_{k} {c_{jk}^{\text{EB}} q_{jk} } }}{{\sum\nolimits_{k} {q_{jk} } }} .$$

Therefore, the total profit function of hospital *k* for providing HRG *j* is:

6 $$\pi_{jk} = R_{jk}^{\text{IN}} - C_{jk}^{\text{IN}} + R_{jk}^{\text{EB}} - C_{jk}^{\text{EB}} = \left[ {p_{jk}^{\text{IN}} \left( {1 + te_{jk} } \right) - c_{jk}^{\text{IN}} } \right]y_{jk} + \left[ {p_{jk}^{\text{EB}} \left( {1 + te_{jk} } \right) - c_{jk}^{\text{EB}} } \right]q_{jk} .$$

The profit margin, i.e., the profit per patient allocated to HRG *j* in hospital *k*, can be written as:

7 $$\tilde{\pi }_{jk} = \frac{{\pi_{jk} }}{{y_{jk} }} = p_{jk}^{\text{IN}} \left( {1 + te_{jk} } \right) - c_{jk}^{\text{IN}} + \left[ {p_{jk}^{\text{EB}} \left( {1 + te_{jk} } \right) - c_{jk}^{\text{EB}} } \right]\frac{{q_{jk} }}{{y_{jk} }} ,$$

where $p_{jk}^{\text{IN}} \left( {1 + te_{jk} } \right) - c_{jk}^{\text{IN}}$ is the *inlier* profit margin of hospital *k* for HRG *j*, and $p_{jk}^{\text{EB}} \left( {1 + te_{jk} } \right) - c_{jk}^{\text{EB}}$ is the *per diem* profit margin of hospital *k* for each excess bed day produced under HRG *j*. As prices are fixed, this simply demonstrates that profitability will vary according to differences in costs that are not accounted for in the payment arrangement.^7^

Several factors driving hospital unit costs may also explain differences between specialist and general hospitals. Following Bradford et al. [24], we summarise these in the following function:

8 $$c_{jk} = c\left( {\varvec{x}_{jk} ,\varvec{z}_{k} } \right) ,$$

where $\varvec{x}_{jk}$ is a vector of patient characteristics not captured by the HRG classification system; and $\varvec{z}_{k}$ is a vector of hospital characteristics, such as input prices that are not captured fully by the market forces adjustment, teaching activity, or economies of scale. For instance, specialist hospitals are likely to employ surgeons with advanced expertise that are paid higher salaries, and to use more costly high-tech equipment. A high level of specialisation is likely to produce high quality of care and, perhaps, higher costs. Specialist hospitals might attract higher volumes of patients, which may allow them to exploit economies of scale but could translate into larger proportions of complex patients requiring a more intensive use of resources. Below, in our empirical analysis, we are able to control for a number of such explanatory factors.

## Econometric specification

We focus on four key specifications. The dependent variable is the log of the *inlier* unit cost ($c_{jk}^{\text{IN}}$) or the *per diem* unit cost ($c_{jk}^{\text{EB}}$).^8^ All models are estimated by WLS in order to take into account, respectively, the number of patients ($y_{jk}$) or excess bed days ($q_{jk}$) of every HRG within each hospital. Moreover, we cluster standard errors within hospitals in order to allow for any form of serial correlation of errors across HRGs.

In the first regression, model I, we test whether unit costs are on average higher in specialist orthopaedic hospitals before accounting for any differences in payments across hospitals:

9 $$\ln \left( {c_{jk} } \right) = \mu + \beta s_{k} + \varepsilon_{jk} ,$$

where $c_{jk}$ is the *inlier* or *per diem* unit cost of HRG *j* in hospital *k*, *μ* is the intercept, $s_{k}$ is a dummy equals one if hospital *k* is a specialist orthopaedic hospital, and $\varepsilon_{jk}$ is the error term.

The estimated coefficient $\hat{\beta }$ translates into $\tilde{\beta } = \exp \left( {\hat{\beta }} \right) - 1$ [25, p. 240; 26]. This expresses the percentage difference in unit costs between specialist orthopaedic hospitals and T&O departments in general hospitals, i.e., $\tilde{\beta } = {{\left( {\bar{c}_{\text{s}} - \bar{c}_{\text{g}} } \right)} \mathord{\left/ {\vphantom {{\left( {\bar{c}_{\text{s}} - \bar{c}_{\text{g}} } \right)} {\bar{c}_{\text{g}} }}} \right. \kern-0pt} {\bar{c}_{\text{g}} }}$ with $\bar{c}_{\text{s}}$ and $\bar{c}_{\text{g}}$ being respectively the specialist orthopaedic hospitals and the T&O departments’ unit cost averaged across HRGs and hospitals. Suppose that $\tilde{\beta } > 0$, which implies higher unit costs in specialist orthopaedic hospitals. This, however, does not necessarily imply that specialist orthopaedic hospitals have lower profit margins because no account is taken of hospital revenue. Specialist orthopaedic hospitals may provide more expensive treatments that are fully compensated by a higher HRG tariff.

Our second and main econometric specification, model II, accounts for differences in payments across specialist orthopaedic hospitals and T&O departments:

10 $$\ln \left( {c_{jk} } \right) = \mu + \beta s_{k} + \gamma m_{k} + \delta e_{jk} + \alpha_{j} + \varepsilon_{jk} ,$$

where $m_{k}$ is the MFF index, $e_{jk}$ is the proportion of specialised services, and $\alpha_{j}$ indicates a set of HRG fixed effects that controls for differences in average cost for each HRG; in turn, this controls for the fixed prices at HRG level, which are based on the average cost within each HRG.

This specification compares unit costs across specialist orthopaedic hospitals and T&O departments, after differences in the MFF and specialist top-up payments are taken into account. The tariffs are subtracted through the HRG fixed effects, i.e., a dummy variable for each HRG *j*. The estimated coefficient of every HRG dummy captures the average unit cost of the corresponding HRG category. Suppose again that $\tilde{\beta } > 0$ (computed using the estimated $\hat{\beta }$ in model II). This result now implies that specialist orthopaedic hospitals exhibit lower profit margins compared with T&O departments.

If we find that specialist orthopaedic hospitals are less financially viable, the finding could be due to a number of competing reasons, which we account for in our model III. Following common practice [e.g., 27, 28], this model controls for patient and hospital characteristics that may explain differences in unit costs in addition to differences in payments and, therefore, profitability:

11 $$\ln \left( {c_{jk} } \right) = \mu + \beta s_{k} + \gamma m_{k} + \delta e_{jk} + \varvec{\rho^{\prime}x}_{jk} + \varvec{\theta^{\prime}z}_{k} + \alpha_{j} + \varepsilon_{jk} ,$$

where $\varvec{x}_{jk}$ is a vector of patient characteristics measured at HRG level namely the proportion of males, average age, average socio-economic status, average number of diagnoses, and procedures; and $\varvec{z}_{k}$ is a vector of hospital characteristics such as doctor salaries, a dummy indicating whether the hospital is teaching hospital or a foundation trust, size dummies calculated using the number of T&O beds to capture potential economies of scale, the average patient outcomes for hip and knee replacement as measure of quality, and regional dummies to allow for residual geographical differences not captured by other adjustments.

The estimated coefficient $\hat{\beta }$ in model II provides an average effect across specialist orthopaedic hospitals. There may be heterogeneity in terms of their financial position, with some exhibiting lower deficits and others higher surpluses. To explore such heterogeneity, as a sensitivity analysis, we estimate the following model IV, which includes hospital fixed effects and directly standardises unit costs ($c_{jk}$) by the MFF index ($m_{k}$):

12 $$\ln \left( {{{c_{jk} } \mathord{\left/ {\vphantom {{c_{jk} } {m_{k} }}} \right. \kern-0pt} {m_{k} }}} \right) = \mu + \varvec{\beta^{\prime}h}_{k} + \delta e_{jk} + \alpha_{j} + \varepsilon_{jk} .$$

In this specification, the specialist orthopaedic hospital dummy ($s_{k}$) used in model I, II, or III is replaced with a vector of hospital dummies ($\varvec{h}_{k}$). Also $\varvec{\beta}$ is now a vector including *k* coefficients, one for each hospital dummy: for instance, if $\hat{\beta }_{k} > 0$ then the provision of trauma and orthopaedic services in hospital *k* implies lower profit margins relative to the average hospital. We directly standardise unit costs ($c_{jk}$) because the MFF index ($m_{k}$) would be perfectly collinear with hospital dummies ($\varvec{h}_{k}$) if added as an additional control variable.

All regression models are estimated separately for *inlier* and *per diem* unit costs because the HRG price is computed separately for *inlier* and *excess bed day* activity. For each model, we obtain the *inlier* and *per diem* estimates of $\beta$, which are then used to compute an *overall* measure of cost (for model I) or profitability (for models II, III, and IV). For instance, consider our key model II in Eq. (10), which estimates the percentage difference in *inlier* or *per diem* profit margins between specialist orthopaedic hospitals and T&O departments. The percentage difference in *overall* profit margin per patient treated between specialist orthopaedics hospitals and T&O departments, after allowing for differences in unit costs of excess bed days, can be written as:

13 $$\frac{{\bar{\pi }_{\text{g}} - \bar{\pi }_{\text{s}} }}{{\bar{C}_{\text{g}} }} = \frac{{\left( {\bar{c}_{\text{s}}^{\text{IN}} - \bar{c}_{\text{g}}^{\text{IN}} } \right)\bar{y} + \left( {\bar{c}_{\text{s}}^{\text{EB}} - \bar{c}_{\text{g}}^{\text{EB}} } \right)\bar{q}}}{{\bar{c}_{\text{g}}^{\text{IN}} \bar{y} + \bar{c}_{\text{g}}^{\text{EB}} \bar{q}}} ,$$

where $\bar{\pi }_{\text{g}} - \bar{\pi }_{\text{s}}$ is the difference in profit averaged across HRGs and hospitals between T&O departments and specialist orthopaedic hospitals, expressed as a percentage of the T&O departments’ total cost averaged across HRGs and hospitals, $\bar{C}_{\text{g}}$ (to be consistent with the interpretation of profitability of the *inlier* activity, $\tilde{\beta }^{\text{IN}}$, and *excess bed day* activity, $\tilde{\beta }^{\text{EB}}$); $\bar{c}_{s}^{IN} - \bar{c}_{g}^{IN} = \tilde{\beta }^{IN} \bar{c}_{g}^{IN}$ and $\bar{c}_{\text{s}}^{\text{EB}} - \bar{c}_{\text{g}}^{\text{EB}} = \tilde{\beta }^{\text{EB}} \bar{c}_{\text{g}}^{\text{EB}}$ are the difference in *inlier* and *per diem* unit costs averaged across HRGs and hospitals, respectively; $\bar{y}$ and $\bar{q}$ are the average volume of patients and the average number of excess bed days, respectively.^9^ Standard errors of the *overall* estimates are bootstrapped using 1000 replications.

## Data

Our primary source of data is the RC database for the financial year 2013/14. For every admission type of every single inpatient HRG, each hospital annually reports information on *inlier* unit costs, *per diem* unit costs, number of patients, and excess bed days.

Hospitals follow a standard process in calculating unit costs by applying the rules set out in the NHS costing manual, which establishes three basic principles [29]: first, costs capture the full cost of the services delivered, so that they can be reconciled back to the original aggregated costs in the accounts; second, costs are preferably allocated through direct imputation rather than through apportionment; and third, costs rigorously match the services generating them. The costing process consists of a top-down approach that, in the first instance, groups total costs into: costs that are directly attributable to patients (e.g., doctors, nurses, drugs); costs that are only indirectly linked to patients and that are identified on an activity basis (e.g., linen, catering); and overhead costs that are not related to patients (e.g., senior managers, administrative employees). Such costs are then attributed to macro-areas of treatment and support services (e.g., pharmacy, building maintenance), to hospital specialties (e.g., general surgery, orthopaedics), to wards, and finally to HRGs. Costs are further split by admission type such as non-elective (short or long), elective, and day case.^10^ Cost data are audited and must comply with validation rules to assure their accuracy, which is fundamental for the calculation of the national tariffs [30].

Our sample for the analysis of *inlier* unit costs consists of 79,096 observations across 1284 HRGs and 134 hospitals.^11^ Of these observations, 14,181 refer to day case treatment, 18,170 to elective care, 19,532 are short-stay non-elective care, and 27,186 are long-stay non-elective care. The sample for the analysis of *per diem* unit costs comprises 16,098 observations, of which 4087 are elective and 12,011 are non-elective.

For every HRG in each hospital, we calculate the proportion of patients who receive specialised orthopaedic services, the proportion of male patients, average patient age, average socio-economic status, average number of diagnoses, and procedures using data summarised from patient-level information in the HES [31].^12^ We collect several variables measured at hospital level, most of which are from the Health and Social Care Information Centre (HSCIC; since renamed NHS Digital): a dummy variable for specialist orthopaedic hospitals, teaching hospitals, and foundation trusts; the average salary of doctors employed in the T&O specialty;^13^ and regional dummies. The HSCIC also provides Patient Reported Outcome Measures (PROMs) including, for each hospital, the average health change of patients undergoing hip and knee replacement [32, 33]. PROMs measure the patients’ quality of life through the EQ-5D health-status questionnaire before and 6 months after their surgery. Hence, the health change is the difference between the post and pre-surgery EQ-5D scores, and it is estimated through a risk-adjustment methodology that takes account of patient characteristics and factors beyond hospitals’ control [34].^14^ Using data from the NHS statistics, we construct dummies related to the size of the T&O department (small, medium, large, and very large), which are defined on the quartiles of the T&O beds distribution of all hospitals. Finally, the RC database reports the MFF index.

### Descriptive statistics

**Fig. 1:**
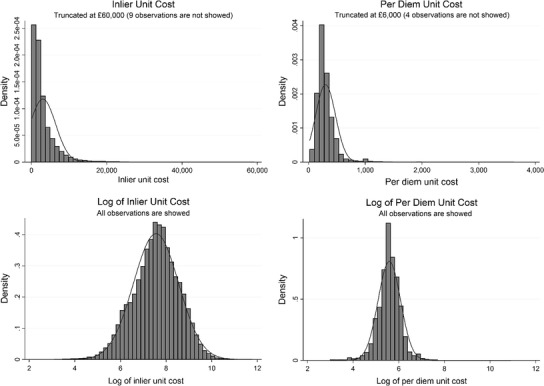
Distribution of *inlier* and *per diem* unit costs in natural units and logs

Figure 1 illustrates that the distribution of *inlier* and *per diem* unit costs substantially departs from normality when in natural units, while it is approximately normal after taking the log. Table 1 contains descriptive statistics of the variables measured at HRG level for the sample with observations of all admission types.^15^ Our sample includes the three specialist orthopaedic hospitals and 131 T&O departments in general hospitals. Specialist orthopaedic hospitals have on average higher *inlier* unit costs than T&O departments (£5196 vs. £2987) and a higher number of patients per HRG (20 vs. 12). The proportion of patients receiving specialised services is higher in specialist orthopaedic hospitals (1.1%) than T&O departments (0.1%). 49% of patients are male in both specialist orthopaedic hospitals and T&O departments, while patients in specialist orthopaedic hospitals are on average 8 years younger (47 vs. 55) and better-off (deprivation index greater by 2%). Specialist orthopaedic hospitals record about the same number of diagnoses (5) for their average patient but provide one more procedure (4 vs. 3) than T&O departments.

**Table 1:** Descriptive statistics of variables measured at HRG level

Variable at HRG level	All hospitals				Specialist hospitals				General hospitals			
	Mean	SD	Min	Max	Mean	SD	Min	Max	Mean	SD	Min	Max
Inlier												
Inlier unit cost	3031	3484	22	129,419	5196	8555	173	129,419	2987	3287	22	78,447
Number of patients (FCEs)	12.2	37.4	1	1622	20.3	57.7	1	644	12.1	36.9	1	1622
Number of specialised services	0.05	0.73	0	55	0.66	4.23	0	55	0.04	0.42	0	26
Proportion of specialised services (%)	0.1	1.7	0.0	100.0	1.1	6.1	0.0	69.2	0.1	1.5	0.0	100.0
Proportion of males (%)	49.1	19.6	0.0	100.0	49.2	24.3	0.0	100.0	49.1	19.5	0.0	100.0
Age	54.7	18.9	0.0	97.0	47.4	17.4	1.0	90.0	54.8	18.9	0.0	97.0
Deprivation index	15,969	4889	12	32,474	16,296	4365	194	32,417	15,963	4899	12	32,474
Number of diagnoses	4.969	2.655	1	20	4.733	2.511	1	13	4.974	2.657	1	20
Number of procedures	3.079	2.108	0	24	4.118	2.158	0	12	3.058	2.102	0	24
Number of HRGs	1284				415				1272			
Observations	79,069				1564				77,505			
Excess bed day												
Per diem unit cost	305	474	20	54,422	465	2867	65	54,422	301	188	20	9499
Number of excess bed days	22.2	35.5	1	715	44.8	81.8	1	715	21.7	33.4	1	538
Number of specialised services	0.11	1.31	0	55	1.95	7.56	0	55	0.07	0.56	0	26
Proportion of specialised services (%)	0.2	2.0	0.0	69.2	2.7	9.6	0.0	69.2	0.1	1.3	0.0	45.6
Proportion of males (%)	46.8	16.2	0.0	100.0	47.5	18.1	0.0	100.0	46.8	16.2	0.0	100.0
Age	57.8	15.7	0.1	97.0	49.2	16.2	7.9	90.0	58.0	15.6	0.1	97.0
Deprivation index	16,047	4564	201	32,268	16,499	3636	1428	31,664	16,036	4583	201	32,268
Number of diagnoses	5.096	2.535	1	20	4.906	2.501	2	13	5.100	2.536	1	20
Number of procedures	3.160	2.096	0	24	4.378	2.265	0	12	3.131	2.084	0	24
Number of HRGs	675				183				662			
Observations	16,098				373				15,725			

The lower part of Table 1 also provides descriptive statistics for excess bed days. *Per diem* unit costs are on average higher in specialist orthopaedic hospitals (£465) than in T&O departments (£301). There are on average 22 excess bed days per HRG, but many more in the specialist orthopaedic hospitals (45) than in T&O departments (22). The proportion of patients receiving specialised services with a *per diem* unit cost is also higher in specialist orthopaedic hospitals (2.7% vs. 0.1%). Similarly, the proportion of male patients with a long length of stay in specialist hospitals is slightly greater than in T&O departments (47.5% vs. 46.8%). Long-stay patients are 9 years younger (49 vs. 58), better-off (deprivation index greater by 3%), and have the same number of diagnoses (5) but one more procedure (4 vs. 3) in specialist orthopaedic hospitals compared to T&O departments.

Table 2 illustrates descriptive statistics of the variables measured at hospital level. 24 (17.9%) trusts are teaching hospitals, and 80 (59.7%) hospitals have foundation status. Two of the specialist orthopaedic hospitals are foundation trusts but none is a teaching hospital. 15 hospitals are in the London region, one of which is specialised. The remaining two specialist orthopaedic hospitals are located in the West Midlands region, which includes 14 other general hospitals. The regions with the largest and smallest number of hospitals are, respectively, the North West including 22 hospitals, and the East Midlands and the North East with 8 hospitals. On the basis of the quartile division, a T&O department is categorised as small if it has less than 46 specialty beds, medium if between 46 and 61 beds, large if between 62 and 79 beds, and very large if it has more than 79 beds. The three specialist orthopaedic hospitals fall into the very large group. The MFF index is on average greater in specialist orthopaedic hospitals compared to T&O departments (1.085 vs. 1.075). A doctor working in T&O earns on average approximately £86,000. Doctors in specialist orthopaedic hospitals are paid 5.6% more, on average, than doctors in T&O departments.

**Table 2:** Descriptive statistics of variables measured at hospital trust level

Variable at hospital trust level	All hospitals				Specialist hospitals				General hospitals			
	Mean	SD	Min	Max	Mean	SD	Min	Max	Mean	SD	Min	Max
Specialist orthopaedic hospital	0.022	0.148	0	1	1.000	0.000	1	1	0.000	0.000	0	0
Market forces factor	1.076	0.064	1.003	1.298	1.085	0.082	1.032	1.180	1.075	0.063	1.003	1.298
Salary of doctors (£10,000)	8.664	0.744	6.596	10.060	9.134	0.293	8.797	9.324	8.653	0.749	6.596	10.060
Teaching hospital	0.179	0.385	0	1	0.000	0.000	0	0	0.183	0.388	0	1
Foundation hospital	0.597	0.492	0	1	0.667	0.577	0	1	0.595	0.493	0	1
Small department	0.201	0.403	0	1	0.000	0.000	0	0	0.206	0.406	0	1
Medium department	0.284	0.452	0	1	0.000	0.000	0	0	0.290	0.456	0	1
Large department	0.254	0.437	0	1	0.000	0.000	0	0	0.260	0.440	0	1
Very large department	0.261	0.441	0	1	1.000	0.000	1	1	0.244	0.431	0	1
Average health change after hip replacement	0.425	0.028	0.311	0.476	0.442	0.033	0.410	0.476	0.425	0.028	0.311	0.474
Average health change after knee replacement	0.315	0.028	0.215	0.396	0.317	0.025	0.288	0.332	0.315	0.028	0.215	0.396
London	0.112	0.316	0	1	0.333	0.577	0	1	0.107	0.310	0	1
East Midlands	0.060	0.238	0	1	0.000	0.000	0	0	0.061	0.240	0	1
East of England	0.127	0.334	0	1	0.000	0.000	0	0	0.130	0.337	0	1
North East	0.060	0.238	0	1	0.000	0.000	0	0	0.061	0.240	0	1
North West	0.164	0.372	0	1	0.000	0.000	0	0	0.168	0.375	0	1
South East	0.149	0.358	0	1	0.000	0.000	0	0	0.153	0.361	0	1
South West	0.112	0.316	0	1	0.000	0.000	0	0	0.115	0.320	0	1
West Midlands	0.119	0.325	0	1	0.667	0.577	0	1	0.107	0.310	0	1
Yorkshire and The Humber	0.097	0.297	0	1	0.000	0.000	0	0	0.099	0.300	0	1
Number of trusts	134				3				131			

Of all NHS patients treated in the T&O specialty, 9.5% received a hip replacement and 6.7% underwent a knee replacement. Specialist orthopaedic hospitals have a higher average health gain for hip (0.442 vs. 0.425) and knee (0.317 vs. 0.315) replacement.

## Results

Table 3 provides the estimation results of models I, II, and III for *inlier* and *per diem* unit costs when all admission types are included in the sample. Recall that unit costs are in logs. The specialist orthopaedic hospital dummy’s estimated coefficient is positive and statistically significant at 5% level in models I and II but it is insignificant in model III for the *inlier* unit costs. It is always negative but statistically insignificant in the regressions for the *per diem* unit costs. Specialist orthopaedic hospitals and T&O departments in general hospitals have therefore statistically different costs for the *inlier* activity but statistically similar costs for the *excess bed day* activity. The first column of Table 3 shows the estimates of model I in Eq. (9), indicating raw differences in unit costs between specialist orthopaedic hospitals and T&O departments. Specialist orthopaedic hospitals have on average [exp(0.149) − 1 =]^16^ 16.1% higher *inlier* unit costs. In contrast, they have on average 14.4% lower *per diem* unit costs, but this result is not statistically significant.

**Table 3:** Estimation results when all admission types are included

Regressor	Inlier			Per diem		
	Model I	Model II	Model III	Model I	Model II	Model III
Specialist orthopaedic hospital	0.149**	0.185**	0.149	− 0.156	− 0.276	− 0.140
	(0.059)	(0.076)	(0.097)	(0.187)	(0.196)	(0.204)
Market forces factor		0.845***	0.928**		0.353	0.485
		(0.213)	(0.460)		(0.381)	(1.228)
Proportion of specialised services		0.012**	0.010*		0.003	0.003
		(0.005)	(0.006)		(0.003)	(0.003)
Proportion of males			− 0.00009			− 0.0004
			(0.000)			(0.001)
Age			− 0.015***			− 0.006
			(0.004)			(0.006)
Age (squared)			0.0001***			0.0001*
			(0.000)			(0.000)
Deprivation index			− 0.000003			− 0.000007
			(0.000)			(0.000)
Number of diagnoses			0.037***			− 0.031*
			(0.010)			(0.018)
Number of procedures			0.024***			− 0.017
			(0.007)			(0.012)
Salary of doctors			0.003			− 0.041
			(0.021)			(0.040)
Teaching trust			0.057*			0.097
			(0.034)			(0.076)
Foundation trust			− 0.049*			0.011
			(0.026)			(0.059)
Medium department			− 0.019			− 0.068
			(0.035)			(0.081)
Large department			− 0.021			0.002
			(0.032)			(0.083)
Very large department			0.022			− 0.117
			(0.034)			(0.077)
Average health change after hip replacement			0.952*			− 1.896*
			(0.523)			(1.081)
Average health change after knee replacement			− 0.414			0.468
			(0.465)			(1.177)
Constant			6.625***			6.429***
			(0.608)			(1.607)
HRG fixed effects	NO	YES	YES	NO	YES	YES
Regional fixed effects	NO	NO	YES	NO	NO	YES
Observations	79,069	79,069	79,069	16,098	16,098	16,098
Adjusted* R*^2^	0.001	0.797	0.805	0.005	0.074	0.157

Standard errors are clustered at hospital trust level and are reported in parentheses

***p value < 0.01, **p value < 0.05, *p value < 0.1

Model II in Eq. (10) provides estimates of differences in unit costs after accounting for differences in revenue by subtracting tariffs (HRG fixed effects) and by accounting for tariff adjustments (MFF and specialised services top-ups). The specialist orthopaedic hospital dummy’s estimated coefficient therefore can be interpreted as the difference in profit margins between specialist orthopaedic hospitals and T&O departments.^17^ Specialist orthopaedic hospitals have on average 20.3% lower *inlier* profit margins. A percentage point increase in the proportion of specialised services raises *inlier* unit costs by 1.2%. A standard deviation increase in the MFF (0.064) is associated with an increase in *inlier* unit costs of 5.6%.

With model III in Eq. (11), we investigate whether differences in profit margins can be explained by patient and hospital characteristics. The differences in *inlier* and *per diem* unit costs ($\hat{\beta }$) are both statistically insignificant, as are the variables capturing hospital characteristics. Instead, patient characteristics measuring age and number of diagnoses and procedures are significant at 1% level in explaining the differences in *inlier* (but not *per diem*) profit margins between specialist orthopaedic hospital and T&O departments.^18^ Age and *inlier* unit costs have a quadratic relationship so that unit costs decrease up to 75 years (− 0.015/(2 × 0.0001)) and increase above that. At the sample mean of 54.7 years, one more year decreases* inlier* unit costs by 0.4% (− 0.015 + 2 × 0.0001 × 54.7). An additional diagnosis or procedure raises *inlier* unit costs by 3.8% or 2.4%, respectively. We extend model III by adding interactions between all control variables. We find that differences in both *inlier* and *per diem* profit margins between specialist orthopaedic hospitals and T&O departments remain statistically insignificant (see Table 10 in the "Appendix 3").^19^

So far, we have presented our findings on specialist orthopaedic hospitals for *inlier* and *excess bed day* hospital activity, separately. Table 4 reports the *overall* percentage change in unit costs ($\tilde{\beta }$) between specialist orthopaedic hospitals and T&O departments for each admission type.^20^ The *overall* percentage change is calculated as the sum of *inlier* and *per diem* percentage changes in unit costs or profit margins. The first column shows the percentage changes derived from model I. The *overall* unit costs are not statistically different between specialist orthopaedic hospitals and T&O departments. In model II, when hospital revenues are taken into account, specialist orthopaedic hospitals have on average 13% lower *overall* profit margins than T&O departments at 1% of statistical significance (see footnote 17 for details on the interpretation). Model III shows that the *overall* profit margins in specialist orthopaedic hospitals are no longer significantly different from those in T&O departments after controlling for some key determinants including patient characteristics such as proportion of males, age, socio-economic status, number of diagnoses and procedures, and hospital characteristics such as salary of doctors, hospital type, specialty size, quality, and other regional differences.

**Table 4:** Differences in unit costs between specialist orthopaedic hospitals and T&O departments in general hospitals

Inpatient activity	Model I	Model II	Model III
All admission types			
Overall^a^	0.114	0.135***	0.116
	(0.157)	(0.000)	(0.466)
Inlier	0.161**	0.203**	0.161
	(0.013)	(0.016)	(0.125)
Per diem	− 0.144	− 0.241	− 0.131
	(0.408)	(0.161)	(0.494)
Elective			
Overall^a^	0.254***	0.226***	0.204**
	(0.000)	(0.000)	(0.026)
Inlier	0.311***	0.282***	0.249***
	(0.000)	(0.000)	(0.000)
Per diem	− 0.225	− 0.248	− 0.176
	(0.195)	(0.175)	(0.243)
Long non-elective			
Overall^a^	0.601***	0.389***	0.403*
	(0.000)	(0.000)	(0.076)
Inlier	0.741*	0.499***	0.486***
	(0.064)	(0.004)	(0.003)
Per diem	− 0.140	− 0.192	− 0.033
	(0.395)	(0.196)	(0.864)
Short non-elective	0.293	0.320	0.369*
	(0.101)	(0.147)	(0.099)
Day case	− 0.071	0.029	− 0.018
	(0.731)	(0.887)	(0.924)

^a^Standard errors are bootstrapped using 1000 replications

*p* value in parentheses; ***p value < 0.01, **p value < 0.05, *p value < 0.1

### Sensitivity analysis

As a sensitivity analysis, we estimate the same three models for each admission type. The lower panel of Table 4 (second column) shows that statistically significant lower *overall* profit margins in specialist orthopaedic hospitals are found for elective (22.6%) and long non-elective activity (38.9%), but not for short non-elective and day case activity.

**Fig. 2:**
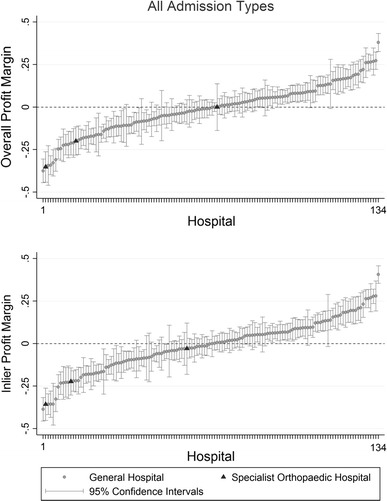
Distribution of *overall* and *inlier* profit margins

Finally, estimation of model IV including hospital fixed effects in Eq. (12) suggests wide variation in *overall* (*inlier*) profit margins across hospitals in our sample, from 37.5% (38.6%) below the average to 38% (40.6%) above the average. Figure 2 indicates that 45 hospitals, i.e., about a third, have significantly lower *overall* profit margins compared to the average profit margins, and 42 have significantly higher *overall* profit margins.^21^ None of the three specialist orthopaedic hospitals have *overall* or *inlier* profit margins significantly above the average. In particular, as shown in Table 5, the *overall* profit margins of the Robert Jones and Agnes Hunt orthopaedic hospital (− 19.9%) and the Royal orthopaedic hospital (− 35.2%) are significantly below the average.^22^ The Royal National Orthopaedic Hospital has instead average *overall* profit margins. The latter finding is driven by higher profit margins on day case activity (40.6%).

**Table 5:** Specialist orthopaedic hospitals’ *overall* profit margins

Specialist orthopaedic hospital	All admission types (%)	Day case (%)	Elective (%)	Short non-elective	Long non-elective (%)
Royal National Orthopaedic Hospital NHS Trust	0.0	40.6*	− 30.5*	− 79.4*	− 80.5*
Robert Jones and Agnes Hunt Orthopaedic Hospital NHS Foundation Trust	− 19.9*	− 21.5*	− 18.0*	4.2	− 4.8
Royal Orthopaedic Hospital NHS Foundation Trust	− 35.2*	− 29.5*	− 29.0*	− 69.0 *	− 30.6*

* Significantly different from the average hospital at 5%

## Discussion and conclusions

The English NTPS is used to reimburse hospitals according to the amount and mix of activity that they undertake. Like most PPSs, there is a recognition that HRGs imperfectly account for all patient or exogenous hospital characteristics that might influence costs [35, 36]. As such, payment adjustments include top-ups to the tariff if patients received particular specialised care and payment corrections allow for differential costs of labour and capital across the country. These refinements help ensure a fair reimbursement system that rewards hospitals according to the care that they provide, not the advantageous circumstances in which they might operate [5, 37].

Given these payment adjustments, hospitals that provide care at a cost below tariff should be more profitable. Arguably specialist hospitals should be in a strong position to benefit financially from these arrangements. By focussing on a limited set of services they should be able to better exploit informational or organisational advantages associated with specialisation. Such advantages derive from concentrating on a specific, defined caseload that enhances learning-by-doing and attracts staff with particular expertise and more easily allows efficient practice in care delivery to be identified and operationalised [13].

If these advantages obtain we would expect specialist hospitals to earn higher profits than general hospitals that undertake similar activities. The evidence provided in this study does not support this claim. We have analysed the costs and revenues associated with delivery of trauma and orthopaedic services in all three specialist orthopaedic hospitals and 131 T&O departments in general hospitals in England. We find that, compared to the national average, profit margins are 13% lower in the three specialist orthopaedic hospitals. Profits are statistically significantly lower across all patients that have at least one overnight stay, either elective or non-elective.

These lower profits are not due simply to patients in specialist hospitals requiring long lengths of stay or specialist care. Payment arrangements allow for this possibility through *excess bed day* payments and tariff top-ups for specialised treatments, and we account for these revenue adjustments in our analysis. Nor does it appear that differences can be explained by the characteristics of the hospitals such as their teaching and foundation status or geographical location, nor by the number of the T&O patients treated, nor by variation in doctors’ salaries, nor by the quality of care as captured by PROMs for two high-volume orthopaedic procedures such as hip and knee replacement. Lower profits are observed even after these potential explanatory factors are taken into account.

Instead, we find that lower profit margins in specialist orthopaedic hospitals are explained by patient characteristics such as age and severity as captured by the number of diagnoses and procedures. This means that although hospital payments are based on a detailed patient classification system (HRG) and on adjustments for the higher cost of specialised care, providers that generally attract more complex patients such as specialist orthopaedic hospitals may be financially disadvantaged. That said, being part of a general hospital does not guarantee better financial performance with 33% of the T&O departments also making a loss.

Our study has three main limitations. First, our sample includes only three specialist orthopaedic hospitals. Such a small number of specialist orthopaedic hospital trusts, however, is not the result of sample selection but reflects the reality that there are only three specialist orthopaedic hospital trusts in the English NHS. Specialist hospitals are few and far between in many countries. Hence, we believe that our analysis is appropriate and generally applicable. Moreover, although we are limited by the actual number of hospitals, we analyse hundreds of HRGs for each specialist hospital and we investigate heterogeneity across the three hospitals in model IV using hospital fixed effects. This model shows that two of the three specialist hospitals make a loss and none of them makes a profit, which confirms that specialist orthopaedic hospitals are in a relatively weak financial position.

Second, our estimated tariffs may not be identical to current tariffs, i.e., the actual tariffs that hospitals receive in 2013/14. We compute tariffs by including in our models (II, III, or IV) the HRG fixed effects, which capture the unit cost of each HRG averaged across hospitals. This reflects the methodology used to compute current tariffs but, in practice, current tariffs are based on cost data lagged by 3 years in order to ensure data accuracy and stakeholder engagement in their calculation [21]. To account for the time lag, the current tariffs’ methodology adjusts for inflation and efficiency trends. We therefore argue that tariffs estimated through our methods are a reasonable approximation to current tariffs.

Finally, PROMs are currently available only for two orthopaedic procedures such as hip and knee replacements. These procedures are, however, the most common in T&O departments: of all NHS patients treated in the T&O specialty, 9.5% receive a hip replacement and 6.7% undergo a knee replacement. We therefore argue that hip and knee replacements are indicative of departmental performance.

Future research may be required before a definitive recommendation about whether profit margins differ in trauma and orthopaedic services across general and specialised hospitals. But we have set out a methodology that can be applied to other types of hospital service and in other settings, to investigate the extent to which differences in costs between groups of hospitals are adequately covered by prospective payment systems.

## Figures and Tables

**Fig. 3:**
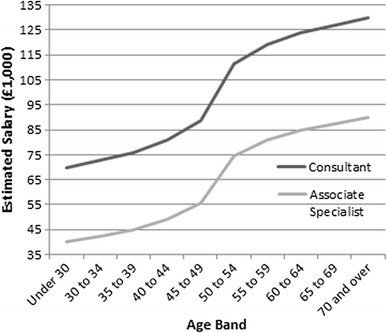
Estimated salary function for consultants and associate specialists

## Appendix 1: estimation of the salary of doctors

We assume that the salary of doctors follows a s-shape function depending on age, minimum and maximum salary. This means that salary rises with increasing returns in the first half of the working life, and it goes up with decreasing returns during the second half. In symbols, we estimate the salary as follows:

14 $$w_{nk} = f_{nk} \cdot W\left( {w^{\hbox{min} } ,w^{\hbox{max} } ,A_{\text{age}} } \right)$$

where $w_{nk}$ is the salary of doctor *n* ($= 1, \ldots ,N$) in hospital *k*, $f_{nk}$ is the full-time equivalent ratio,^23^$W$ is the s-shape salary function, $w^{\hbox{min} }$ and $w^{\hbox{max} }$ are the minimum and maximum salaries associated to the doctor’s grade, and $A_{\text{age}}$ is a coefficient varying depending on the doctor’s age. The salary function $W$ can be represented as follows:

15 $$W\left( {w^{{\min }} ,w^{{\max }} ,A_{{age}} } \right) = \left\{\begin{array}{*{20}l} {w^{{\min }} } \hfill & {{\text{if age}} < 30} \hfill \\ {w^{{\min }} + \frac{{w^{{\max }} - w^{{\min }} }}{{A_{{age}} }}} \hfill & {{\text{ if 30}} \le {\text{age}} < 50} \hfill \\ {w^{{\max }} - \frac{{w^{{\max }} - w^{{\min }} }}{{A_{{age}} }}} \hfill & {{\text{ if 30}} \le {\text{age}} < 50} \hfill \\ {w^{{\max }} } \hfill & {{\text{ if age}} \ge 50} \hfill \\ \end{array} \right.$$

where,

16 $$A_{{{\text{age}}}} = \left\{ {\begin{array}{*{20}c} {20{\text{ }}\,{\text{if 30}} \le {\text{age}} \le 34{\text{ }}\,{\text{or }}\,{\text{65}} \le {\text{age}} \le 69} \\ {10{\text{ }}\,{\text{if 35}} \le {\text{age}} \le 39{\text{ }}\,{\text{or }}\,{\text{60}} \le {\text{age}} \le 64} \\ {5.5{\text{ }}\,{\text{if 40}} \le {\text{age}} \le 44{\text{ }}\,{\text{or }}\,{\text{55}} \le {\text{age}} \le 59} \\ {3.2{\text{ }}\,{\text{if 45}} \le {\text{age}} \le 54{\text{ }}\,{\text{or }}\,{\text{55}} \le {\text{age}} \le 59} \\ \end{array} } \right.$$

In Fig. 3, we illustrate the salary function $W\left( \cdot \right)$ for consultant and associate specialist doctors.

The average salary of doctors in hospital *k* ($w_{k}$) is therefore calculated as follows:

17 $$w_{k} = \frac{{\sum\nolimits_{n} {w_{nk} } }}{{N_{k} }} .$$

## Appendix 2: descriptive statistics by admission type

See Tables 6 and 7.

**Table 6:** Descriptive statistics for day case and elective activity

Variable at HRG level	All hospitals		Specialist hospitals		General hospitals	
	Mean	SD	Mean	SD	Mean	SD
Day case						
Inlier unit cost	1408	876	1492	973	1406	872
Number of patients (FCEs)	26	67	33	77	25	66
Number of specialised services	0.07	0.75	0.43	3.10	0.06	0.52
Proportion of specialised services (%)	0.2	1.8	0.6	4.2	0.2	1.6
Proportion of males (%)	49.8	19.5	48.6	22.5	49.8	19.4
Age	49.6	17.2	44.2	17.3	49.8	17.1
Deprivation index	16,039	4769	16,140	3868	16,036	4795
Number of diagnoses	3.718	1.704	3.983	1.926	3.709	1.695
Number of procedures	3.449	1.630	4.041	1.729	3.430	1.623
Number of HRGs	509		239		490	
Observations	14,181		441		13,740	
Elective						
Inlier						
Inlier unit cost	3680	3620	5978	8808	3586	3200
Number of patients (FCEs)	16	42	23	58	15	41
Number of specialised services	0.07	0.94	0.61	4.06	0.05	0.47
Proportion of specialised services (%)	0.2	2.3	1.2	6.3	0.2	1.9
Proportion of males (%)	48.9	19.5	49.4	26.3	48.9	19.2
Age	54.6	16.9	47.6	17.9	54.8	16.8
Deprivation index	16,080	4807	16,368	4648	16,068	4813
Number of diagnoses	4.644	2.369	4.908	2.640	4.633	2.357
Number of procedures	3.516	1.901	4.195	2.288	3.488	1.879
Number of HRGs	730		350		696	
Observations	18,179		716		17,463	
Excess bed day						
Per diem unit cost	358	897	563	3450	344	245
Number of excess bed days	19	34	49	92	17	25
Number of specialised services	0.24	1.91	1.65	6.66	0.14	0.88
Proportion of specialised services (%)	0.4	2.9	2.5	9.0	0.3	1.8
Proportion of males (%)	46.7	14.4	46.6	18.1	46.7	14.1
Age	56.2	13.3	48.9	16.3	56.7	12.9
Deprivation index	16,235	4350	16,557	3762	16,213	4386
Number of diagnoses	4.343	2.076	4.807	2.494	4.312	2.041
Number of procedures	3.656	1.838	4.416	2.263	3.605	1.795
Number of HRGs	313		151		282	
Observations	4087		257		3830

**Table 7:** Descriptive statistics for short non-elective and long non-elective activity

Variable at HRG level	All hospitals		Specialist hospitals		General hospitals	
	Mean	SD	Mean	SD	Mean	SD
Short non-elective						
Inlier unit cost	1253	1381	2154	3412	1248	1358
Number of patients (FCEs)	6	12	2	1	6	12
Number of specialised services	0.03	0.39	0.20	1.93	0.03	0.36
Proportion of specialised services (%)	0.1	0.9	0.4	3.8	0.1	0.8
Proportion of males (%)	49.5	18.6	50.0	21.9	49.5	18.6
Age	52.7	20.8	47.8	16.8	52.7	20.8
Deprivation index	15,908	4843	15,869	4634	15,908	4845
Number of diagnoses	4.840	2.562	4.490	2.196	4.842	2.564
Number of procedures	2.466	1.907	3.656	2.115	2.459	1.903
Number of HRGs	839		97		836	
Observations	19,523		119		19,404	
Long non-elective						
Inlier						
Inlier unit cost	4720	4241	10,181	12,150	4661	4035
Number of patients (FCEs)	8	17	3	3	8	18
Number of specialised services	0.04	0.76	1.31	6.28	0.03	0.37
Proportion of specialised services (%)	0.1	1.7	1.9	8.4	0.1	1.4
Proportion of males (%)	48.6	20.5	49.4	22.6	48.5	20.4
Age	58.9	18.7	51.4	15.7	59.0	18.7
Deprivation index	15,902	5035	16,529	4240	15,895	5043
Number of diagnoses	5.933	2.947	5.547	2.770	5.937	2.949
Number of procedures	3.035	2.458	4.235	2.403	3.022	2.456
Number of HRGs	1022		175		1020	
Observations	27,186		288		26,898	
Excess bed day						
Per diem unit cost	286	162	247	148	287	162
Number of excess bed days	23	36	35	53	23	36
Number of specialised services	0.07	1.02	2.61	9.25	0.04	0.40
Proportion of specialised services (%)	0.1	1.6	3.2	10.8	0.1	1.1
Proportion of males (%)	46.9	16.8	49.6	18.1	46.8	16.8
Age	58.4	16.4	49.8	15.9	58.5	16.4
Deprivation index	15,983	4633	16,370	3353	15,979	4643
Number of diagnoses	5.352	2.625	5.126	2.512	5.354	2.626
Number of procedures	2.992	2.151	4.296	2.276	2.979	2.146
Number of HRGs	647		86		643	
Observations	12,011		116		11,895	

## Appendix 3: additional sensitivity analysis

See Tables 8, 9, 10 and 11.

**Table 8:** Stepwise regression analysis in model III for *inlier* unit costs

Regressor	Inlier							
	(1)	(2)	(3)	(4)	(5)	(6)	(7)	(8)
Specialist orthopaedic hospital	0.147	0.199**	0.200**	0.178**	0.181**	0.159*	0.165*	0.170*
	(0.099)	(0.085)	(0.085)	(0.089)	(0.083)	(0.095)	(0.086)	(0.091)
Market forces factor	0.810*	0.947**	0.945**	0.875**	1.043**	0.927**	1.027**	1.036**
	(0.450)	(0.441)	(0.441)	(0.435)	(0.469)	(0.459)	(0.468)	(0.463)
Proportion of specialised services	0.010*	0.011**	0.011**	0.010*	0.010*	0.010*	0.010*	0.011**
	(0.005)	(0.005)	(0.005)	(0.006)	(0.006)	(0.006)	(0.006)	(0.005)
Proportion of males	0.00005		0.0004	− 0.0001	− 0.0001	− 0.00004	− 0.0002	0.0004
	(0.000)		(0.000)	(0.000)	(0.000)	(0.000)	(0.000)	(0.000)
Age	− 0.015***			− 0.013***	− 0.013***	− 0.014***	− 0.014***	
	(0.004)			(0.004)	(0.004)	(0.004)	(0.004)	
Age (squared)	0.00008**			0.00007*	0.00008*	0.00009**	0.0001**	
	(0.000)			(0.000)	(0.000)	(0.000)	(0.000)	
Deprivation index	− 0.000003				− 0.000004	− 0.000003	− 0.000004	− 0.000004
	(0.000)				(0.000)	(0.000)	(0.000)	(0.000)
Number of diagnoses	0.041***					0.042***		0.033***
	(0.011)					(0.010)		(0.010)
Number of procedures	0.028***						0.032***	0.027***
	(0.007)						(0.008)	(0.007)
Salary of doctors		− 0.001	− 0.001	− 0.001	− 0.0006	0.003	− 0.0007	0.003
		(0.021)	(0.021)	(0.020)	(0.020)	(0.021)	(0.020)	(0.021)
Teaching trust		0.081**	0.081**	0.070**	0.066*	0.058*	0.063*	0.066*
		(0.036)	(0.037)	(0.036)	(0.035)	(0.034)	(0.035)	(0.035)
Foundation trust		− 0.063**	− 0.063**	− 0.060**	− 0.058**	− 0.050*	− 0.055**	− 0.052**
		(0.026)	(0.026)	(0.026)	(0.026)	(0.026)	(0.026)	(0.026)
Medium department		− 0.007	− 0.007	− 0.013	− 0.021	− 0.017	− 0.022	− 0.015
		(0.035)	(0.035)	(0.035)	(0.036)	(0.035)	(0.036)	(0.035)
Large department		− 0.013	− 0.013	− 0.018	− 0.021	− 0.021	− 0.021	− 0.018
		(0.034)	(0.034)	(0.033)	(0.033)	(0.032)	(0.033)	(0.033)
Very large department		0.026	0.026	0.022	0.02	0.023	0.019	0.024
		(0.034)	(0.034)	(0.034)	(0.035)	(0.034)	(0.034)	(0.034)
Average health change after hip replacement		0.825	0.825	0.847	0.89	0.952*	0.900*	0.936*
		(0.554)	(0.553)	(0.546)	(0.546)	(0.524)	(0.540)	(0.531)
Average health change after knee replacement		− 0.519	− 0.515	− 0.485	− 0.373	− 0.453	− 0.332	− 0.402
		(0.474)	(0.474)	(0.477)	(0.476)	(0.467)	(0.473)	(0.466)
Constant	7.100***	6.372***	6.356***	6.913***	6.697***	6.681***	6.618***	5.979***
	(0.553)	(0.589)	(0.592)	(0.589)	(0.607)	(0.603)	(0.611)	(0.612)
HRG fixed effects	YES	YES	YES	YES	YES	YES	YES	YES
Regional fixed effects	YES	YES	YES	YES	YES	YES	YES	YES

Standard errors are clustered at hospital trust level and are reported in parentheses

****p* value < 0.01, ***p* value < 0.05, **p* value < 0.1

**Table 9:** Stepwise regression analysis in model III for *per diem* unit costs

Regressor	Per diem							
	(1)	(2)	(3)	(4)	(5)	(6)	(7)	(8)
Specialist orthopaedic hospital	− 0.131	− 0.107	− 0.131	− 0.123	− 0.102	− 0.101	− 0.096	− 0.112
	(0.304)	(0.295)	(0.304)	(0.306)	(0.278)	(0.282)	(0.278)	(0.278)
Market forces factor	0.490	0.910	0.497	0.529	1.239	1.246	1.234	1.133
	(1.274)	(1.144)	(1.271)	(1.244)	(1.357)	(1.334)	(1.356)	(1.371)
Proportion of specialised services	0.000	0.001	0.000	0.000	0.001	0.001	0.001	0.000
	(0.004)	(0.004)	(0.004)	(0.004)	(0.004)	(0.004)	(0.004)	(0.004)
Proportion of males		− 0.0007	− 0.0009	− 0.0006	− 0.0005	− 0.0005	− 0.0005	− 0.001
		(0.001)	(0.001)	(0.001)	(0.001)	(0.001)	(0.001)	(0.001)
Age		− 0.011		− 0.010	− 0.011	− 0.011	− 0.010	
		(0.013)		(0.014)	(0.014)	(0.013)	(0.014)	
Age (squared)		0.000		0.000	0.000	0.000	0.000	
		(0.000)		(0.000)	(0.000)	(0.000)	(0.000)	
Deprivation index		− 0.000010			− 0.00002*	− 0.00002*	− 0.00002*	− 0.00002
		(0.000)			(0.000)	(0.000)	(0.000)	(0.000)
Number of diagnoses		0.006				− 0.002		0.002
		(0.029)				(0.029)		(0.029)
Number of procedures		− 0.022					− 0.012	− 0.018
		(0.021)					(0.021)	(0.020)
Salary of doctors	− 0.068		− 0.068	− 0.068	− 0.065	− 0.065	− 0.065	− 0.065
	(0.049)		(0.049)	(0.049)	(0.047)	(0.047)	(0.047)	(0.047)
Teaching trust	0.014		0.015	0.020	0.006	0.006	0.007	− 0.001
	(0.087)		(0.087)	(0.089)	(0.089)	(0.091)	(0.090)	(0.090)
Foundation trust	0.045		0.045	0.045	0.052	0.051	0.050	0.050
	(0.071)		(0.071)	(0.070)	(0.070)	(0.068)	(0.070)	(0.069)
Medium department	− 0.065		− 0.064	− 0.061	− 0.095	− 0.095	− 0.095	− 0.098
	(0.088)		(0.088)	(0.089)	(0.096)	(0.096)	(0.096)	(0.096)
Large department	0.069		0.07	0.073	0.057	0.057	0.057	0.052
	(0.094)		(0.094)	(0.097)	(0.099)	(0.099)	(0.099)	(0.098)
Very large department	− 0.036		− 0.036	− 0.032	− 0.043	− 0.043	− 0.043	− 0.048
	(0.092)		(0.092)	(0.092)	(0.090)	(0.091)	(0.090)	(0.092)
Average health change after hip replacement	− 0.225		− 0.222	− 0.228	− 0.031	− 0.036	− 0.028	− 0.020
	(1.275)		(1.274)	(1.276)	(1.282)	(1.283)	(1.280)	(1.283)
Average health change after knee replacement	0.936		0.924	0.895	1.392	1.397	1.375	1.382
	(1.211)		(1.215)	(1.222)	(1.255)	(1.267)	(1.255)	(1.275)
Constant	5.539***	4.979***	5.568***	5.732***	4.810***	4.810***	4.863***	4.854**
	(1.744)	(1.350)	(1.752)	(1.647)	(1.753)	(1.754)	(1.759)	(1.922)
HRG fixed effects	YES	YES	YES	YES	YES	YES	YES	YES
Regional fixed effects	YES	YES	YES	YES	YES	YES	YES	YES

Standard errors are clustered at hospital trust level and are reported in parentheses

****p* value < 0.01, ***p* value < 0.05, **p* value < 0.1

**Table 10:** Analysis of interactions between covariates in model III

Regressor	Inlier		Per diem
Specialist orthopaedic hospital	0.083		− 0.146
(1) Market forces factor	− 6.022		− 1.440
(2) Proportion of specialised services	− 0.198*		− 0.054
(3) Proportion of males	− 0.005		− 0.012
(4) Age	0.044		− 0.004
(5) Age (squared)	− 0.001***		0.000
(6) Deprivation index	0.000		0.000
(7) Number of diagnoses	0.116		0.064
(8) Number of procedures	− 0.123		0.022
(9) Salary of doctors	− 0.614		− 1.054
(10) Teaching trust	− 1.071		− 1.821
(11) Foundation trust	− 0.712		− 2.798***
(12) Medium department	0.100		4.843***
(13) Large department	1.150		2.900
(14) Very large department	1.099		4.579**
(15) Average health change after hip repl.	0.656		13.009
(16) Average health change after knee repl.	− 0.082		− 27.838
Interactions			
(1) × (2)	0.150**	(4) × (10)	0.010**
(3) × (2)	0.0002**	(6) × (2)	0.000002***
(3) × (9)	− 0.001**	(6) × (13)	− 0.00002**
(5) × (11)	0.0001**	(6) × (14)	− 0.00005***
(5) × (4)	0.00001***	(6) × (15)	0.001***
(7) × (2)	− 0.003**	(7) × (2)	0.002**
(7) × (3)	− 0.0004**	(7) × (5)	− 0.00004**
(8) × (11)	0.026**	(8) × (11)	0.029***
(8) × (4)	− 0.003***	(11) × (9)	− 0.167**
(8) × (5)	0.00003***	(12) × (1)	− 3.635***
(13) × (11)	− 0.184***	(13) × (11)	0.398***
(14) × (2)	0.028***	(14) × (1)	− 3.815***
(15) × (2)	− 0.18**	(15) × (12)	− 5.829**
(15) × (11)	2.199**	(16) × (9)	3.314***
(15) × (12)	− 2.634**	(16) × (11)	8.573***
(15) × (14)	3.088**	(16) × (12)	5.422**
(16) × (14)	− 3.108**		
Constant	13.516**		12.454
HRG fixed effects	YES		YES
Regional fixed effects	YES		YES
Adjusted *R*^2^	0.814		0.307

Interactions not significant at 1% or 5% level are not reported

Standard errors are clustered at hospital trust level

****p* value < 0.01, ***p* value < 0.05, **p* value < 0.1

**Table 11:** Results for model V that includes hospital random effects

Regressor	Inlier	Per diem
Specialist orthopaedic hospital	0.122	− 0.201
	(0.342)	(0.288)
Market forces factor	0.428	0.049
	(0.651)	(1.488)
Proportion of specialised services	0.014*	0.002
	(0.008)	(0.003)
Proportion of males	− 0.0006	− 0.0003
	(0.000)	(0.001)
Age	− 0.018***	− 0.006
	(0.004)	(0.006)
Age (squared)	0.0001***	0.00008
	(0.000)	(0.000)
Deprivation index	− 0.000001	− 0.000003
	(0.000)	(0.000)
Number of diagnoses	0.040***	− 0.016
	(0.012)	(0.012)
Number of procedures	0.013	− 0.015*
	(0.009)	(0.008)
Salary of doctors	0.008	− 0.044
	(0.030)	(0.048)
Teaching trust	0.076	0.108
	(0.051)	(0.086)
Foundation trust	− 0.052	0.03
	(0.037)	(0.071)
Medium department	0.006	− 0.063
	(0.051)	(0.090)
Large department	− 0.015	0.041
	(0.047)	(0.098)
Very large department	0.008	− 0.121
	(0.049)	(0.088)
Average health change after hip replacement	1.345*	− 2.202
	(0.768)	(1.361)
Average health change after knee replacement	− 0.137	0.003
	(0.691)	(1.389)
Constant	− 0.050***	− 0.024
	(0.018)	(0.031)
Hospital random effects	YES	YES
HRG fixed effects	YES	YES
Regional fixed effects	YES	YES

Maximum likelihood estimation. For ease of computation, we control for the HRG fixed effects using the Frisch–Waugh–Lovell transformation

Clustered standard errors are bootstrapped with 1000 repetitions and are reported in parentheses

****p* value < 0.01, ***p* value < 0.05, **p* value < 0.1

## Footnotes

1 There are specialist hospitals in Europe [4, 6], America [7, 8], Asia [9], India [10], and Africa [11].

2 More than 60% of hospital income comes from the NTPS. The remaining part is agreed in the NHS standard contract on the basis of actual activity [19].

3 The trim point is the maximum expected length of stay for a patient falling under a specific HRG. It is defined by the Department of Health in order to identify unusually long lengths of stay and statistical outliers [21].

4 At the time of our study, top-ups were paid for children’s, orthopaedic, spinal, and neurosciences specialised services. While all hospitals can obtain the top-up for specialised orthopaedic services, top-ups for the other specialised services are paid to a restricted number of eligible providers.

5 In the English NHS, a hospital trust or acute trust is an authority that provides secondary health care services through one or more acute hospitals.

6 The number of patients is expressed by the number of finished consultant episodes (FCEs). A FCE is a hospital episode for a patient under the care of an individual consultant.

7 To illustrate this point, suppose that a specialist orthopaedic hospital *s* and a T&O department in general hospital *g* have the same volume of patients and excess bed days ($y_{\text{s}} = y_{\text{g}}$, $q_{\text{s}} = q_{\text{g}}$), the same location ($m_{\text{s}} = m_{\text{g}}$), and the same proportion of top-up tariffs ($e_{\text{s}} = e_{\text{g}}$). Then, differences in profits will be equal to $\pi_{\text{g}} - \pi_{\text{s}} = \left( {c_{\text{s}}^{\text{IN}} - c_{\text{g}}^{\text{IN}} } \right)y_{\text{g}} + \left( {c_{\text{s}}^{\text{EB}} - c_{\text{g}}^{\text{EB}} } \right)$. For instance, $\pi_{\text{g}} - \pi_{\text{s}} > 0$ implies that the specialist orthopaedic hospital has lower profit margins than the T&O department in a general hospital. Such a difference will reflect factors not allowed for in the payment mechanism.

8 We take the logarithm to improve model fit, since unit costs are left-skewed. All estimated coefficients are therefore interpreted as semi-elasticities.

9 The computation of the *overall* profitability for model IV in Eq. (12) differs from the computation described in Eq. (13). It becomes ${{\left( {\bar{\pi }_{k} - \bar{\pi }} \right)} \mathord{\left/ {\vphantom {{\left( {\bar{\pi }_{k} - \bar{\pi }} \right)} {\bar{C}}}} \right. \kern-0pt} {\bar{C}}} = {{\left[ {\left( {\bar{c}_{k}^{\text{IN}} - \bar{c}^{\text{IN}} } \right)\bar{y} + \left( {\bar{c}_{k}^{\text{EB}} - \bar{c}^{\text{EB}} } \right)\bar{q}} \right]} \mathord{\left/ {\vphantom {{\left[ {\left( {\bar{c}_{k}^{\text{IN}} - \bar{c}^{\text{IN}} } \right)\bar{y} + \left( {\bar{c}_{k}^{\text{EB}} - \bar{c}^{\text{EB}} } \right)\bar{q}} \right]} {\left( {\bar{c}^{\text{IN}} \bar{y} + c^{\text{EB}} \bar{q}} \right)}}} \right. \kern-0pt} {\left( {\bar{c}^{\text{IN}} \bar{y} + c^{\text{EB}} \bar{q}} \right)}}$, where $\bar{\pi }_{k}$ and $\bar{c}_{k}$ are the hospital *k*’s total profit and unit cost respectively averaged across HRGs and hospitals, $\bar{\pi }$ and $\bar{c}$ are the total profit and unit cost respectively averaged across HRGs and hospitals, $\bar{C}$ is the total cost averaged across HRGs and hospitals, $\left( {\bar{c}_{k}^{\text{IN}} - \bar{c}^{\text{IN}} } \right) = \tilde{\beta }^{\text{IN}} \bar{c}^{\text{IN}}$ and $\left( {\bar{c}_{k}^{\text{EB}} - \bar{c}^{\text{EB}} } \right) = \tilde{\beta }^{\text{EB}} \bar{c}^{\text{EB}}$. Also in this case, the standard errors of the *overall* estimates are bootstrapped using 1000 repetitions.

10 Unlike elective and day case patients, the admission of non-elective patients is unplanned. Day case and short non-elective patients do not have an overnight stay in hospital, while elective and long non-elective patients have at least one overnight stay.

11 10 T&O departments in general hospitals did not report data on PROMs for hip or knee replacement and they are, therefore, dropped from the sample.

12 We count specialised services following the rules defined in the Prescribed Specialised Services, and not the criteria specified in the Specialised Services National Definition Sets. We use the overall Index of Multiple Deprivation (IMD) as a measure of socio-economic status. This index is constructed through the combination of seven IMD domains such as income, employment, health deprivation and disability, education, skills and training, barriers to housing and services, crime, and living environment. A value of one indicates extreme deprivation while 32,482 indicates no deprivation.

13 The salary of a doctor employed in the T&O specialty is estimated through a s-shape function of age, minimum and maximum salary. Further details are provided in the "Appendix 1".

14 More precisely, the risk-adjustment methodology comprises three steps. The first step consists of estimating a generalised least-square fixed-effects model in which the dependent variable is the post-surgery EQ-5D score of each patient, the covariates are pre-surgery EQ-5D score, patient characteristics (e.g., gender, age, ethnicity), economic deprivation, comorbidity, procedure and post-operative length of stay. This regression also controls for unobserved hospital heterogeneity through fixed effects. In the second step, the model is used to estimate predictions. The third step aggregates such predictions to obtain the adjusted average post-surgery EQ-5D score for each provider, from which the national average pre-surgery EQ-5D score is subtracted for the calculation of the adjusted average health gain.

15 Tables 6 and 7 in the "Appendix 2" show descriptive statistics of the variables measured at HRG level for the sample with day case and elective observations, and short non-elective and long-non elective observations, respectively.

16 The exponential transformation is applied to all the figures reported in the text in this section. This explains the differences with the coefficients reported in Table 3.

17 Recall that the unit cost is the dependent variable in model II (III or IV) while tariffs are on the right-hand side of the equation. Under such a regression design, *β* reflects the difference between unit costs and tariffs instead of the definition of profit margins, i.e., difference between tariffs and unit costs. To abide by the correct definition of profit margins, the interpretation of *β* must be reversed so that, for example, a positive estimate indicates lower profit margins in specialist orthopaedic hospitals relative to T&O departments in general hospitals.

18 To reinforce this finding, we provide the results of a stepwise regression in Table 8 in the "Appendix 3". These results show that age, number of diagnoses, and procedures together drive the differences in *inlier* unit costs between specialist orthopaedic hospitals and T&O departments in general hospitals, with there being a seeming difference between the hospital types if any of these patient characteristics are omitted. Table 9 shows that differences between hospital types in *per diem* unit costs are always statistically insignificant whether or not patient characteristics are accounted for.

19 As a further robustness check, we estimate model V, which is akin to model III but also includes hospital random effects. Unlike the hospital fixed-effects model, the hospital random-effects model can be estimated when the specialist orthopaedic hospital dummy is included, although this requires the additional assumption that the covariates are uncorrelated with the HRG-invariant unobserved hospital heterogeneity. Table 11 in the "Appendix 3" shows that the results for model V are very similar to those for model III.

20 Recall that the percentage change ($\tilde{\beta }$) is obtained through the exponential transformation of the estimated coefficient ($\hat{\beta }$).

21 We count only hospitals for which confidence intervals do not overlap the dashed horizontal line at zero, i.e., hospitals for which the deviation of profit margins from the mean is statistically different from zero.

22 Recall that *β*_*k*_ in model IV captures the deviation of hospital *k*’s profit margins from the mean profit margins: a positive *β*_*k*_ means that hospital *k*’s profit margins are lower than the mean, while a negative *β*_*k*_ suggests that hospital *k*’s profit margins are higher than the mean (see also footnote 17 for details on the interpretation). For ease of interpretation, we multiply the estimate of *β*_*k*_ by minus one and, therefore, the negative sign now indicates profit margins that are lower than the mean. All coefficients in Table 5 indicate the percentage change ($\tilde{\beta }_{k}$) obtained through the exponential transformation of $\hat{\beta }_{k}$.
